# South West Paediatric Club

**Published:** 1992-03

**Authors:** 


### South West Paediatric Club

### Meeting held at the State House Hotel, Land's End, 27 April 1991

### THE PAINTER AND CORNISH MINERS

Ben Maile
International Artist, Truro

The Cornish miners of the 19th century travelled length and breadth of the USA, not only opening up valuable mines of silver, gold and other precious metals but creating local colonies of the Cornish blooded people who are still there today keeping alive the old Cornish customs.

In preparing a BBC Television programme, based upon second generation Cornish in the USA, Ben Maile travelled over 20,000 miles to areas such as North Michigan, Missouri, Wisconsin, Texas, Arizona, California and Nevada. His paintings and television film depicted old ghost towns in the desert and on mountain tops, all of them at one time, either opened or developed by the expertise of the much sought after "Cousin Jack" — the Cornishman's nickname worldwide.

The presentation shows that, though the mines may now be past industries, the persistent, adventurous Cornishman has established proud colonies of descendants who, today, keep alive all the old Cornish customs and celebrations. Their one ambition is now to visit or revisit their homeland.

### CHILDHOODS OF THE FAMOUS

Sheila Eades FRCP
Consultant Paediatrician
Royal Cornwall Hospital, Truro

Paediatricians believe that the environment and upbringing of a child shapes his future as an adult. A study of the childhood of some who achieved greatness shows that many were underprivileged, sickly, had difficult births and suffered from physical handicap. A significant number were orphaned early or had poor relationships with their mothers.

Genius was seldom recognised in childhood. The quality is difficult to define. Schopenhauer said that it is "The ability to see a pattern in things and to see the general in the particular". Meyers defined genius as "The power of utilising a wider range than other men of faculties to some degree innate in all".

Examples were given from the lives of Winston Churchill, Mozart, Montgomery of Alamein, John Wesley, Florence Nightingale, Van Gogh, Einstein, the Bronte family and others who achieved lasting fame.

### LESSONS FROM THE WHOOPING COUGH VACCINE TRIAL

Cameron Bowie, MRCP, FFPH
Director of Public Health
Somerset Health Trust

The judgement contradicts the established medical view that pertussis vaccine causes permanent brain damage, albeit rarely. The judge rejected the series of cases which caused the concern in the first place and emphasised the power of selection bias. He could find no evidence to establish a causal link between the vaccine and brain damage. All the biological mechanisms suggested by the plaintiff were improbable with febrile convulsions least so, and the judge concluded that the National Childhood Encephalopathy study was the only way to prove an association. However, he found the study design biased towards vaccine damage and serious errors in the data, sufficient to upturn the results.

The cost of the pertussis following the reduced immunisation uptake over the twelve year period from 1974-85, was an additional half a million pertussis cases, of which about 20,000 required hospital admissions and some sixty cases died. There will have been untold additional morbidity. The need to prevent a similar immunisation programme failure requires an improved surveillance system of adverse reactions to vaccines. The system needs to be of sufficient power and reliability to be able to reassure the public and the medical professional alike. A pilot study was proposed to be carried out in the South Western Region. This requires active case finding in a prospective and controlled study to identify severe neurological adverse reactions to childhood vaccination. After debate, the meeting supported the idea of such a study.

### TRUST THE NHS

Mary Lindsey MB, Ch.B, MRCPsych
Consultant in Mental Handicap
Cornwall and Isles of Scilly Mental Handicap Trust

The Cornwall and Isles of Scilly Mental Handicap NHS Trust had been in existence since April 1991 when the Medical Director of the Trust gave her talk in May to the South West Paediatric Club on her personal experiences in the saga of setting up the Trust. Her view that, for some health services, Trust status offers a valuable opportunity for change, was pragmatic rather than idealistic and based on the particular situation of their services. It is important to understand the psychology of change and to be sure that the reactions to Trusts are not veiled self-interest or fear of change. It is even harder to find the facts and the hidden agendas.

The process which went on, from the decision to apply through to acceptance and the preparation of the business plan, was also described. It served as a fascinating new experience and induction into a new world of business jargon and protocol which is to be recommended to anyone who wants a change from seeing patients and who enjoys watching politics and network dynamics in action. Participation can be stressful and a well developed sense of cynicism offers a protective advantage.

### PAEDIATRIC EXPERIENCE AND TRAINING IN AUSTRALIA

Lisa Goldsworthy MRCP
Paediatric Registrar
Royal Cornwall Hospital, Truro

The experiences gained by spending a year of registrar training at the Royal Children's Hospital, Brisbane were discussed.

The late Dr. David Fraser had, with Professor Baum (Bristol) and Dr. S. Eades (Truro), assisted in arranging what had been planned as an exchange of registrars. The geographical and demographic distribution of Queensland, its capital and the capital's children's hospitals were outlined as well as the differences in health service provision with particular reference to private practice commitments of the consultant specialists with only a couple of full-time hospital appointments. The similarities in medical care were highlighted.

Differences in junior hospital doctor training, principally relating to shorter hours and a "shift" system were explained though the potential problems of limited experience and hand over quality illustrated. In general, a year in another country should prove to be a broadening and highly rewarding experience.

**THE PLACE FOR PAEDIATRICIANS** (Separate article)

